# Burden of Pneumococcal Disease in Young Children Due to Serotypes Contained in Different Pneumococcal Conjugate Vaccines in Eight Asian Countries and Territories

**DOI:** 10.3390/vaccines12101197

**Published:** 2024-10-19

**Authors:** Liping Huang, Xiuyan Li, Ng Eugenia, Johnnie Leung, Sheng-Tzu (Alice) Hung, Ervin Zhi Bin Cheong, Ricardo Avila, Winniefer Nua, Kornvipa Choowanich, Ritika Rampal, Namrata Kulkarni, Derek Daigle, Bulent Nuri Taysi

**Affiliations:** 1 Pfizer, Inc., Collegeville, PA 19426, USA; 2 Pfizer Hong Kong, Quarry Bay, Hong Kong; 3 Pfizer Taiwan, Taipei City 110, Taiwan; 4 Pfizer Singapore, Singapore 117372, Singapore; 5 Pfizer Malaysia, Kuala Lumpur 59200, Malaysia; 6 Pfizer Philippines, Metro Manila 1209, Philippines; 7 Pfizer Thailand, Bangkok 10500, Thailand; 8 Pfizer India Ltd., Mumbai 400051, India

**Keywords:** pneumococcal disease, pneumococcal vaccines, immunization programs, costs, child health

## Abstract

Background: Pneumococcal disease (PD) is a major cause of morbidity and mortality in young children in Asia and globally. Pneumococcal conjugate vaccines (PCVs) have significantly reduced the burden of PD when included in pediatric national immunization programs (NIPs). This study estimates the clinical and economic burden of PD due to serotypes contained in different PCVs in children aged < 5 years in eight Asian countries/territories. Methods: Based on published data, a cohort-based decision analytic model was used to estimate annual PD cases, deaths, and direct medical costs associated with serotypes contained in PCV10, PCV13, PCV15, and PCV20. Results: PD incidence rates were lower in regions with PCV13 in their NIP than those without. Serotypes contained in higher but not lower valency PCVs resulted in a significant incremental clinical and economic burden, although the difference between PCV13 and PCV15 serotypes was generally small. Moving from PCV13 to PCV20 was estimated to result in greater clinical and economic burden reductions. Conclusions: This study demonstrates the remaining and incremental burden of PD from PCV10 to PCV20 serotypes in young children in selected Asian regions. Extending NIP access to higher-valency PCVs with broader serotype coverage and improving vaccine uptake will help prevent morbidity and deaths and save healthcare costs.

## 1. Introduction

Pneumococcal disease (PD) is caused by the bacterium *Streptococcus pneumoniae*. Non-invasive forms of PD include acute otitis media (AOM), rhinosinusitis, and non-bacteremic pneumonia [1]. Invasive pneumococcal disease (IPD) is defined as the isolation of *S. pneumoniae* from normally sterile sites and includes sepsis, bacterial meningitis, and bacteremic pneumonia, among other conditions [1]. PD is a major cause of morbidity and mortality in children under five years of age, with an estimated one million children dying of pneumococcal disease globally every year [2,3]. *S. pneumoniae* poses a significant burden in children in Asia in particular [4]; for example, in India, approximately a quarter of deaths in children < 5 years were attributed to IPD in 2015 [5,6].

As of 2020, 100 serotypes of *S. pneumoniae* have been documented [7]. However, only a limited number are responsible for the majority of PD cases; for example, in the United States, before the development of a vaccine against these serotypes, seven commonly isolated serotypes from the cerebrospinal fluid of children 0–4 years old accounted for 80% of infections [7].

Pneumococcal conjugate vaccines (PCVs) contain polysaccharides of several different serotypes, each conjugated to a carrier protein, conferring protection against those serotypes [8]. The number of serotypes included is referred to as valency. The first PCV (PCV7, Prevnar^®^), licensed for use in the United States in 2000, included serotypes 4, 6B, 9V, 14, 18C, 19F, and 23F [7]. PCV10 (Synforix^TM^) and PCV13 (Prevnar 13^®^) were licensed in the US in 2009 and 2010, respectively [9]. PCV10 includes the same serotypes as PCV7 plus serotypes 1, 5, and 7F, whereas PCV13 includes the ten serotypes covered by PCV10 plus serotypes 3, 6A, and 19A [7]. PCV15 (VAXNEUVANCE^TM^) includes serotypes 22F and 33F in addition to those covered by PCV13, and PCV20 (Prevnar 20^®^) includes 8, 10A, 11A, 12F, and 15B in addition to the coverage of PCV15. PCV15 and PCV20 were licensed in the United States in 2022 and 2023, respectively, and are gradually being licensed across Asia [7].

PCVs have significantly reduced the burden of PD in regions where they have been included in pediatric national immunization programs (NIPs) [6,10,11]. However, even in countries with routine PCV vaccination, the disease persists especially disease due to serotypes not covered by the chosen PCV valency. The introduction of higher-valency PCVs is expected to reduce the burden of PD further.

This study estimates the annual clinical and economic burden of PD attributable to serotypes contained in different PCVs in children aged < 5 years in eight Asian countries/territories and explores the relationship of PCV vaccination in practice to the current burden of the disease.

## 2. Methods

A Microsoft Excel-based decision-analytic model was developed to estimate PD’s annual clinical and economic burden due to serotypes contained in PCV10, PCV13, PCV15, and PCV20 by country in children aged < 5 years. The cohort-based model is based on a cohort of children under five years of age and includes age- and country-specific incidence rates of pneumococcal disease outcomes (i.e., IPD, inpatient/outpatient pneumonia, and AOM), which are used to calculate the estimated number of PD cases per year. Disease outcomes are then stratified based on local serotype coverage for each PCV. Thereafter, age- and country-specific associated costs and case fatality rates are applied to PD outcomes to estimate the direct costs and total deaths associated with each disease outcome by PCV product. Finally, total cases, deaths, and direct costs are aggregated to estimate the annual clinical and economic burden of disease in each country over one year. The aspects of burden evaluated were annual case numbers, annual deaths, and annual direct medical costs. This decision analytic model is informed by previously conducted studies and does not contain any new studies with human participants or animals performed by any of the authors.

The first criterion for countries selected to be included in this study was the presence of well-established national population-based pneumococcal surveillance systems or well-represented published data relevant to the PD incidence and direct medical cost. Countries that have implemented PCV10 or PCV13 NIP for children for at least five years at the time of the data search constituted the second criterion. A targeted literature was conducted, and as a result, eight Asian countries/territories (hereafter referred to as regions) were included: Hong Kong, Singapore, Taiwan, Thailand, Malaysia, Philippines, India, and Indonesia. Regions that had implemented a PCV13 pediatric NIP for at least five years with a vaccination rate of at least 80% at the time of data search (Singapore, Hong Kong, and Taiwan) were designated PCV13 regions. The remainder were designated non-PCV13 regions. The burden associated with PCV13, PCV15, and PCV20 serotypes was estimated for all regions. Additionally, the burden associated with PCV10 serotypes was estimated for the non-PCV13 regions.

## 3. Model Description and Review of Inputs

For the model inputs, the most recent local values were obtained where possible; if these were unavailable, the most appropriate values from other sources were used. Supplementary Table S1 shows the incidence rates used for the model inputs. The full list of model inputs, including case fatality rate and direct medical cost per episode of IPD, all-cause hospitalized or non-hospitalized pneumonia, and AOM, are detailed in Supplementary Table S1. Serotype distribution (i.e., the proportion of cases caused by each serotype covered by PCVs 10 to 20) was obtained from national or regional surveillance data for IPD (Table 1) [12,13,14,15,16,17,18,19]. The same distribution was assumed to apply to the other PD conditions analyzed. The incidence rates used for estimating vaccine-type AOM and pneumonia cases were based on the all-cause incidence rate. The proportion of all-cause pneumonia (18%) [20] or AOM (26.4%) [21] cases caused by *S. pneumoniae* was applied to estimate the incidence of pneumococcal AOM and pneumonia and was assumed to be the same for all countries. Pneumococcal pneumonia was divided into hospitalized and non-hospitalized cases, using proportions from the literature [22,23,24,25,26].

**Figure 1:**
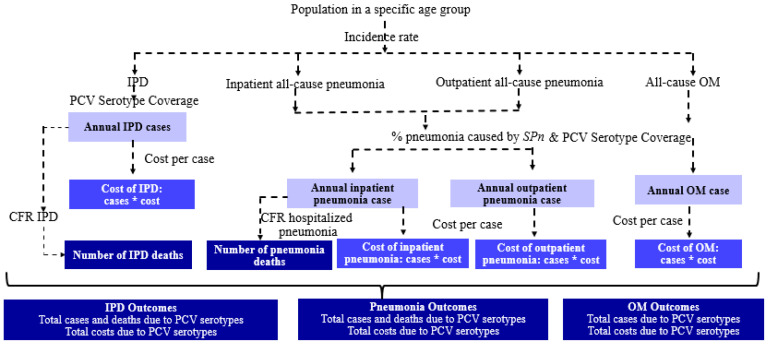
Derivation of clinical and cost outcomes in the model. CFR, case fatality rate; IPD, invasive pneumococcal disease; OM, otitis media; PCV, pneumococcal conjugate vaccine; *SPn*, *Streptococcus pneumoniae*. cases*cost, cases multiplied by cost per case.

The total annual PD cases (IPD, vaccine-type pneumococcal hospitalized and non-hospitalized pneumonia, and AOM) and deaths associated with IPD and hospitalized pneumonia were estimated for children aged < 5 years in each region by applying incidence rates and vaccine serotype coverages to the population size in that age group, taken from official statistics. The number of deaths from each condition was estimated by applying case fatality rates to the case numbers. The total annual direct medical costs were estimated by multiplying the total number of cases of each condition with the estimated cost per episode. All costs were converted and inflated to 2022 USD using country-specific inflation rates. The derivation of clinical and cost outcomes is shown in Figure 1.

## 4. Results

### 4.1. Clinical Burden

The incidence rates used to estimate the burdens of PD were generally lower in the PCV13 regions (Hong Kong, Singapore, and Taiwan) than in the non-PCV13 regions (Thailand, Malaysia, the Philippines, India, and Indonesia) (Supplementary Table S1). The serotype coverage of each PCV ranged from 50–80%, except in Taiwan (Table 1). The serotype coverages of PCV13 and PCV15 were either the same or similar in five regions (Singapore, Taiwan, Thailand, Malaysia, and the Philippines). In non-PCV13 regions, the serotype coverage associated with PCV10 serotypes was the lowest. In India and Indonesia, serotype coverage increased with increasing PCV valency.

The total estimated annual number of PD cases and the estimated cases for each PD condition attributable to serotypes covered by each PCV are shown in Table 2. The total estimated annual deaths are listed in Table 3. Within each country, estimated numbers of PD cases or deaths generally increased along with increasing numbers of serotypes covered by each PCV. The same trend would be expected for vaccine-type pneumococcal AOM and pneumonia.

### 4.2. PCV13 Countries (Hong Kong, Singapore, Taiwan)

Despite including PCV13 in the NIPs, several thousand cases of PD due to vaccine-type serotypes are estimated to occur in each region yearly (Table 2). In Hong Kong, an estimated 7850 cases were associated with PCV13/PCV15 serotypes, and 638 cases were associated with PCV20-unique serotypes. In Singapore, in addition to the 2279 caused by PCV13 serotypes, PCV15 and PCV20-unique serotypes were estimated to cause a further 48 and 463 cases, respectively. In Taiwan, PCV20-unique serotypes could account for an additional 1455 cases beyond 2909 caused by PCV13 or PCV15 serotypes. Notably, there was no difference in the estimated number of cases associated with PCV13 and PCV15 serotypes in Hong Kong or Taiwan and only a very small difference in Singapore. Most PD cases were AOM, but there were appreciable pneumonia cases associated with PCV20 serotypes (an estimated 162 in Hong Kong, 229 in Singapore, and 937 in Taiwan). Very few deaths were estimated to be associated with vaccine-type serotypes in these countries (Table 3).

#### Non-PCV-13 Countries (Thailand, Malaysia, the Philippines, India, and Indonesia)

In these countries, the greater incidences of PD and larger populations resulted in very large numbers of PD cases caused by vaccine-preventable serotypes (Table 2; deaths shown in Table 3), despite PCV10 being part of the NIP in Malaysia, the Philippines, India, and Indonesia. There were more cases from serotypes covered by PCV13 than serotypes covered by PCV10, with particular differences in Thailand, Malaysia, and the Philippines. Thailand had an estimated 12,701 PD cases associated with PCV10 serotypes and a further 4233 and 606 cases from the serotypes covered by PCV13/PCV15 and PCV20-unique serotypes, respectively. There were an estimated 131 deaths from PCV10 serotypes and an additional 47 and six deaths from PCV13/PCV15 and PCV20-unique serotypes, respectively. There was no difference between PCV13 and PCV15 serotypes in either cases or deaths.

Malaysia had an estimated 101,135 cases from PCV10 serotypes, a further 38,615 cases from the additional serotypes in PCV13/PCV15, and a further 2675 from PCV20-unique serotypes. An estimated 266 deaths were due to PCV10 serotypes, with an additional 102 due to PCV13/PCV15 serotypes and a further seven due to PCV20-unique serotypes. There was no difference between PCV13 and PCV15 serotypes in either cases or deaths.

In the Philippines, PD cases amounted to 74,776 and 223,931 caused by PCV10 and PCV13/PCV15 serotypes, respectively. A further 74,379 cases resulted from PCV20-unique serotypes. The number of deaths was 1550 from PCV10 serotypes, an additional 3091 deaths from PCV13/PCV15 serotypes, and a further 1542 from PCV20-unique serotypes. There was estimated to be no difference in cases or deaths due to PCV13 and PCV15 serotypes.

India had by far the largest number of cases and deaths due to PD, reflecting its sizable population. There were an estimated 10.60 million cases of PD from serotypes covered by PCV10, with PCV13, PCV15, and PCV20 incrementally covering an additional 2.05 million, 0.3 million, and 0.78 million, respectively (Table 2). The estimated number of deaths from PCV10 serotypes was 43,161, with an additional 8347 from PCV13 serotypes, 1230 from PCV15 unique serotypes, and 3171 from PCV20-unique serotypes.

In Indonesia, the estimated annual PD case numbers were 846,732 attributable to PCV10 serotypes, 931,405 to PCV13, 1,066,883 to PCV15, and 1,320,902 to PCV20 serotypes. Estimated annual deaths amounted to 5837 attributable to PCV10 serotypes, 6420 to PCV13, 7354 to PCV15, and 9105 to PCV20 serotypes.

### 4.3. Economic Burden

The estimated annual direct medical costs associated with PD caused by PCV13 or PCV15 serotypes in children aged 0–4 years in PCV13 regions ranged from USD 0.11 million in Taiwan to USD 0.70 million in Hong Kong (Table 4). The difference in annual direct medical costs due to PCV13 and PCV15 serotypes was minimal, with no difference estimated in Hong Kong and Taiwan and USD 0.02 million due to PCV15-unique serotypes in Singapore. PCV20-unique serotypes were estimated to result in an additional USD 0.06 million, 0.18 million, and 0.05 million in annual direct medical costs in Hong Kong, Singapore, and Taiwan, respectively.

The estimated annual direct medical costs associated with PD caused by PCV10 serotypes in this age group in the five non-PCV13 regions ranged from USD 3.97 million in Thailand to USD 560.59 million in India. PCV13-unique serotypes were estimated to result in additional annual direct medical costs of USD 1.33 million in Thailand, 30.97 million in Malaysia, 18.38 million in the Philippines, 108.42 million in India, and 8.73 million in Indonesia. The incremental direct medical costs due to additional serotypes contained in PCV15 were USD 15.97 million and USD 13.97 million for India and Indonesia, whereas no additional incremental cost was estimated in Thailand, Malaysia, and the Philippines. The estimated annual incremental direct medical costs due to the five additional serotypes covered in PCV20 were USD 0.19 million in Thailand, 2.14 million in Malaysia, 9.17 million in the Philippines, 41.18 million in India, and 26.19 million in Indonesia.

## 5. Discussion

This study adapts a previously published decision-analytic model to estimate the annual clinical and economic burden of PD attributable to serotypes contained in different PCVs in children aged < 5 years in eight Asian countries/territories. The results highlight that, despite the global success of PCVs, a substantial burden persists in this group associated with serotypes covered by different PCVs in each region studied.

The incidence rates used for the burden estimation indicated that regions that had implemented PCV13 vaccines in their NIPs for at least five years prior to 2020 (Hong Kong, Singapore, and Taiwan) generally had lower incidence rates of PD than those that had not (Malaysia, the Philippines, India, Indonesia, and Thailand). This is likely to be partly due to the greater number of serotypes covered by the PCV13 vaccine, although diagnostic protocols, reporting systems, and social and environmental factors linked to income levels are also likely to affect the incidence rate and serotype coverages used for burden estimation [27,28]. In addition, it may also be influenced by vaccine uptake. However, this study focuses on exploring the PD burdens associated with serotypes covered by PCV10, PCV13, PCV15, and PCV20, not the burdens caused by all serotypes.

Hong Kong first introduced PCV7 into its childhood NIP in 2009, followed by a switch to PCV10 in 2010, PCV13 (3 + 1) in 2011, and PCV13 (2 + 1) in 2019 [29]. In general, PCV uptake in Hong Kong is high: 96.9% of children born in 2014 have received three PCV doses and a booster [30]. Since the introduction of PCV for children, the incidence of IPD has declined significantly in children aged below two years, and a reduction of IPD caused by PCV7 serotypes was observed for all age groups. However, the incidence of IPD caused by PCV13 non-PCV7 serotypes, especially serotype 3, a serotype that is biologically different from other serotypes [31] and emerged as a leading cause of IPD in adults and children in a recent study in Spain [32], has remained high [33,34]. In September 2023, the Scientific Committee on Vaccine Preventable Diseases (SCVPD) recommended replacing PCV13 with PCV15 for use in Hong Kong’s pediatric NIP [33]. The SCVPD noted that further evidence on the clinical effectiveness and impact of PCV15 is still pending [33,35]. As for PCV20, the SVCPD noted that individuals may choose to receive the vaccine to protect themselves against IPD following the manufacturer’s recommendation and discussion with healthcare professionals. Nevertheless, we estimated that 638 cases due to PCV20-unique serotypes could occur each year, whereas no annual increase in PD cases due to PCV15-unique serotypes (22F and 33F) was estimated.

In Singapore, PCV7 was recommended as part of the NIP in 2009, and PCV13 (2 + 1 schedule) was introduced to the NIP in 2011 [36]. The uptake of the final dose in 2022 based on WHO/UNICEF Estimates of National Immunization Coverage (WUENIC) was 90%, up from 52% in 2012 and 84% in 2019 [37]. The disease burdens associated with PCV20 serotypes were higher than PCV13 (2790 vs. 2279 PD cases), and replacing PCV13 with PCV20 could potentially prevent more cases, while with similar PD burdens of PCV15 vs. PCV13 (2327 vs. 2279), the benefit of replacing PCV13 with PCV15 could be minimal.

Taiwan has applied a step-by-step strategy to introduce PCV into the NIP. PCV7 (3 + 1) was first introduced to children younger than five years of age with underlying disease in 2009 and was provided free of charge. It was replaced with PCV10 in 2010, and PCV13 was introduced in 2012 [38,39]. A catch-up program among children 2–5 years of age was chosen as the initial strategy for integrating PCV into the NIP to mitigate the disease burden of IPD among this age group. A universal PCV13 catch-up program was launched in March 2013, providing one dose of PCV13 immunization to children aged 2–5 years. As a result, the incidence of IPD caused by serotypes 3, 6A, and 19F decreased from 11.5 cases per 100,000 person-years in 2012 to 6.2 cases per 100,000 person-years in 2013 within the age group, including a reduction from six to one case/100,000 for serotype 3 [40]. The catch-up program was extended to children aged 1–2 years in January 2014. In January 2015, PCV13 was introduced to the NIP using the 2 + 1 and 3 + 1 schedules for children with and without underlying disease, respectively. This led to a sharp fall in IPD incidence. Uptake was high, with vaccination rates ranging from 90.9% to 99.3% in Taiwanese children under five years of age [41,42]. Our study suggested that, with higher burdens associated with PCV20 serotypes, moving from PCV13 to PCV20 could potentially prevent more cases per year, while moving from PCV13 to PCV15 would provide minimal additional protection as there is no estimated additional disease burden associated with PCV15-unique serotypes (22F and 33F). As a result of the decline in incidence, the Taiwan CDC decided in 2023 to maintain the PCV13 NIP, although PCV15 is available for children in Taiwan [43].

In Thailand, PCV7, PCV10, and PCV13 were licensed between 2007 and 2011, but they have not been included in the National Expanded Programs of Immunization (NEPI) for children as of 2023, although they are available privately [44,45]. No WHO estimates of uptake are available, but it has been suggested that the uptake rate of PCV13 in children in the private market is approximately 13% [46]. According to our analysis, the estimated annual PD cases due to serotypes covered by PCV10 were 12,701. An additional 4233 and 606 PD cases were estimated to be attributable to PCV13-unique serotypes (3, 6A, and 19F) and PCV20-unique serotypes (8, 10A, 11A, 12F, and 15B), respectively. No additional PD burden was estimated to be associated with the two serotypes (22F and 33F) covered in PCV15.

Malaysia included a 3-dose schedule of PCV10 in its pediatric NIP for the first time at the end of 2020, switched to PCV13 (2 + 1 schedule) in March 2023, and expanded coverage to children two to five years old by implementing a catch-up program in May 2023 [47,48]. Before this, private healthcare facilities offered PCV7 and then PCV10 or PCV13. Although uptake following the introduction of a PCV NIP was reported to be 95% for the first dose and 93% for the second dose [47], uptake before the PCV10 NIP in the private market was estimated to be between 15% and 18% of the total yearly birth cohort. Since this study’s epidemiology inputs were collected prior to the implementation of the PCV13 NIP, Malaysia was considered a non-PCV13 country. Our analysis indicated that, with higher burdens associated with PCV13 serotypes, the move from PCV10 to PCV13 could potentially prevent more PD cases. As the additional serotypes covered in PCV15 were estimated to have no incremental PD burden, a move directly from PCV13 to PCV20 (when available) could further prevent additional PD cases attributable to PCV20 serotypes.

In the Philippines, both PCV10 and PCV13 are available. PCV13 was included as a part of the Expanded Program on Immunization (EPI) in selected regions starting in 2015 using a 3 + 0 dosing schedule. According to the 2019 Childhood Immunization Schedule developed by the Philippine Pediatric Society (PPS) and Pediatric Infectious Disease Society of the Philippines (PIDSP), the schedule was revised to 3 + 1 dosing, and catch-up immunizations can be given to children aged fifteen months to five years. Across regions in the Philippines, the vaccination coverage of the 3rd dose of PCV13 for children aged < 1 year ranged from 30–60% for 2015–2019 [49]. Although the uptake rates improved marginally over time, they remained below target at 71% in 2022 (WUENIC final dose estimates) [37,50]. Therefore, it was designated as a non-PCV13 country. Based on the serotype distribution reported in 2020 and incidence rates used in the model inputs, our study suggested that a substantial PD burden remains in the Philippines. An estimated total of 74,776 cases were associated with PCV10 serotypes, and an additional 149,155 cases with the additional three serotypes (3, 6A, and 19A) contained in PCV13. No additional incremental burden was estimated for the two serotypes (22F and 33F) covered in PCV15, but an additional 74,379 PD cases were associated with PCV20-unique serotypes.

In Indonesia, the government experienced challenges in establishing a comprehensive strategy to accelerate the introduction of PCVs. Though intentions for introduction were included in the 2016–2020 comprehensive multiyear plans for immunization (cMYP), there was no official confirmation from the Ministry of Health (MOH) on when and how this would happen. However, the country has received 1.6 million doses of PCV procured through the Gavi Pneumococcal Advanced Market Commitment mechanism, and the PCV immunization Demonstration Program was implemented in 2017–2019 in the West Nusa Tenggara and Bangka Belitung Provinces [51]. The Demonstration Program showed that the PCV immunization coverage during the period was above 80% on average, indicating that PCVs were well received by the community [51]. The official estimate of final dose uptake in 2022 was 39%, though the WUENIC estimate was much lower at 6% [37]. Our analyses estimated that, based on the reported serotype epidemiology, about 0.847, 0.931, 1.067, and 1.321 million cases could occur annually due to serotypes covered by PCV10, PCV13, PCV15, and PCV20, respectively, with the direct medical costs ranging from USD 87.3 million to 136.2 million. Implementing a PCV program, especially a higher-valent PCV, could potentially greatly reduce PD incidence and medical costs.

India introduced PCV10 into the NIP in five states in 2017 [52], with coverage expanded to the remaining states in 2021 [53]. Uptake (WUENIC final dose) rose from 6% in 2018 to 25% in 2021 and 66% in 2022 [37]. Based on the current serotype epidemiology available, we estimated 10.60 million cases attributable to PCV10 serotypes, 12.65 million to PCV13, 12.95 million to PCV15, and 13.73 million to PCV20. PCV10 serotypes were estimated to result in 43,161 deaths, PCV13 in 51,508 deaths, PCV15 in 52,738 deaths, and PCV20 in 55,909 deaths. Moving from a lower-valent to a higher-valent PCV could potentially prevent more disease cases, save lives, and help reduce medical costs.

Overall, the analysis within countries of the incremental cases and deaths from serotypes contained in higher-valency but not lower-valency PCVs highlights that these serotypes result in a significant clinical and economic burden. However, differences in estimated PD cases and deaths between PCV13 and PCV15 serotypes were either not observed, or the differences were relatively small. This suggests that PCV15 may provide little advantage over PCV13, whereas moving directly from PCV13 to PCV20 could substantially reduce clinical and economic burden if uptake was high. The seven unique serotypes contained in PCV20 tend to cause more severe disease and are highly relevant to antimicrobial resistance (11A, 15B, 22F, and 33F), outbreaks (8 and 12F), meningitis (12F, 22F, and 33F), and higher case fatality rate (10A and 11A) compared with other serotypes [54]. While introducing higher-valency PCVs may result in serotype replacement, the overall incidence of PD and its burden will still decline. This has been observed in numerous countries following the introduction of PCV13 into the NIP, including the US, UK, France, and Spain [32,55,56,57].

Our study has several limitations. First, we only studied IPD, pneumococcal pneumonia, and AOM, so other manifestations of PD, such as sinusitis and conjunctivitis, are not taken into account. Second, incidence rates used for estimating cases and deaths are likely to be underestimated as not all PD cases are diagnosed, laboratory-confirmed, serotyped, or reported. For example, Hui et al. recently noted that the burden of IPD in Hong Kong is severely underestimated because of underdiagnosis [58]. This, in turn, means that our estimates of direct medical costs are likely to be conservative. Third, disease in young children carries a significant societal burden from lost productivity and other indirect costs, as parents may have to miss work to look after the child and attend medical appointments. This, together with the cost of medical care, may strain family finances and negatively affect other children. Investigating the wider societal burden would be valuable in creating a more accurate representation of the impact of PD in a region. Fourth, there is significant heterogeneity between the countries and regions studied in terms of population size, economic status, and the quality, accessibility, and cost of healthcare. Thus, while within-region comparisons of the estimated burden from serotypes covered by different vaccines are valuable, between-region comparisons should be treated with caution. Additionally, due to limited data on serotype epidemiology for non-invasive PD disease manifestations, the study assumed that the serotype coverage observed from IPD was also applicable to non-invasive PD, which may not be accurate.

The objective of this study was to capture the remaining burden associated with serotypes covered by lower- and higher-valency PCVs, and it was, therefore, not designed to estimate the potential vaccine impact in terms of cases, deaths, and costs associated with vaccination with these PCVs. Estimating the vaccine’s impact on the disease would involve making assumptions about vaccine efficacy, effectiveness or impact, and the incidence rate and serotype coverages for vaccinated and unvaccinated study populations. Although real-world evidence has demonstrated that PCV10 and PCV13 have substantially impacted PD [59], no efficacy and effectiveness data are yet available for higher-valent PCVs (PCV15 and PCV20). However, based on the disease impact observed from PCV10 and PCV13, we would expect that higher-valent PCVs would further reduce the burden of the PD. Lastly, it is important to be aware that the impact of any PCV program depends on maintaining high vaccine uptake.

## 6. Conclusions

This study demonstrates the remaining incremental clinical and economic burden of PD in children aged 0–4 years associated with serotypes covered by PCV10, PCV13, PCV15, and PCV20 in eight Asian regions. Extending access to higher-valency PCVs with broader serotype coverage in NIPs is expected to prevent substantial morbidity and deaths in young children in Asia and save healthcare costs.

## Figures and Tables

**Table 1:** Serotype coverage for IPD.

	PCV10	PCV13	PCV15	PCV20
**PCV13 regions**				
Hong Kong ^a^	NA	75.9%	75.9%	82.1%
Singapore ^b^	NA	52.1%	53.2%	63.8%
Taiwan ^c^	NA	12.5%	12.5%	18.8%
**Non-PCV13 regions**				
Thailand ^d^	67.7%	90.3%	90.3%	93.6%
Malaysia ^e^	60.5%	83.6%	83.6%	85.2%
Philippines ^f^	18.8%	56.3%	56.3%	75.0%
India ^g^	66.7%	79.6%	81.5%	86.4%
Indonesia ^h^	50.0%	55.0%	63.0%	78.0%

NA, not applicable; PCV, pneumococcal conjugate vaccine. See the Section 1 for a list of the serotypes covered by each vaccine valency. ^a^ Hong Kong Center for Health Protection—Average 2017–2019 [19]; ^b^ Singapore MOH Communicable Disease Surveillance 2018 [12]; ^c^ Taiwan Centers for Disease Control, Project No. MOHW109-CDC-C-315-114116 [13]; ^d^ Thailand MOH Vaccine preventable infectious surveillance 2013–2016, Data on file; ^e^ Lister et al. 2021; ^f^ Philippines Antimicrobial Resistance Surveillance Reference Laboratory Report, 2022 [18]; ^g^ Varghese et al. 2021 [16]; ^h^ Hadinegoro et al. 2016 [17].

**Table 2:** Estimated annual PD cases by serotypes covered by each PCV, by region, in children aged 0–4 years.

	PCV13 Regions			Non-PCV13 Regions				
	Hong Kong	Singapore	Taiwan	Thailand	Malaysia	Philippines	India	Indonesia
Population aged 0–4 years	228,994	178,085	811,733	2,275,366	2,612,247	11,066,707	346,149,367	22,414,317
Total PD cases *								
PCV10	NA	NA	NA	12,701	101,135	74,776	10,599,314	846,732
PCV13	7850	2279	2909	16,934	139,750	223,931	12,649,256	931,405
PCV15	7850	2327	2909	16,934	139,750	223,931	12,951,185	1,066,883
PCV20	8488	2790	4364	17,540	142,425	298,310	13,729,846	1,320,902
**IPD cases**								
PCV10	NA	NA	NA	192	711	2663	41,097	8966
PCV13	15	4	2	256	983	7975	49,045	9862
PCV15	15	4	2	256	983	7975	50,216	11,297
PCV20	16	5	3	265	1002	10,624	53,235	13,987
**Pneumonia cases**								
PCV10	NA	NA	NA	10,063	12,058	19,549	3,123,828	504,322
PCV13	150	187	625	13,417	16,662	58,542	3,727,987	554,754
PCV15	150	191	625	13,417	16,662	58,542	3,816,972	635,446
PCV20	162	229	937	13,897	16,981	77,987	4,046,458	786,743
**AOM cases**								
PCV10	NA	NA	NA	2446	88,366	52,564	7,434,388	333,444
PCV13	7686	2088	2283	3261	122,105	157,414	8,872,224	366,789
PCV15	7686	2132	2283	3261	122,105	157,414	9,083,998	420,140
PCV20	8310	2556	3424	3378	124,442	209,699	9,630,152	520,173

NA, not applicable/not available; PCV, pneumococcal conjugate vaccine; PD, pneumococcal disease, * IPD, AOM, and pneumococcal pneumonia only.

**Table 3:** Estimated annual PD deaths attributable to serotypes covered by each PCV, by region, in children aged 0–4 years.

Total PD Deaths *	PCV13 Regions			Non-PCV13 Regions				
	Hong Kong	Singapore	Taiwan	Thailand	Malaysia	Philippines	India	Indonesia
PCV10	NA	NA	NA	131	266	1550	43,161	5837
PCV13	1	1	0	178	368	4641	51,508	6420
PCV15	1	1	0	178	368	4641	52,738	7354
PCV20	2	1	0	184	375	6183	55,909	9105

NA, not applicable or not available; PCV, pneumococcal conjugate vaccine; PD, pneumococcal disease. * Due to IPD and hospitalized pneumonia cases.

**Table 4:** Estimated annual direct medical costs associated with PD attributable to serotypes covered by each PCV in children aged 0–4 years (reported in millions of 2022 USD).

	PCV13 Regions			Non-PCV13 Regions				
	Hong Kong	Singapore	Taiwan	Thailand	Malaysia	Philippines	India	Indonesia
PCV10	NA	NA	NA	3.97	81.10	9.22	560.59	87.29
PCV13	0.70	0.90	0.11	5.30	112.07	27.60	669.01	96.02
PCV15	0.70	0.92	0.11	5.30	112.07	27.60	684.98	109.99
PCV20	0.76	1.10	0.16	5.49	114.21	36.77	726.16	136.18

NA, not applicable or not available; PCV, pneumococcal conjugate vaccine.

## Data Availability

The original data used for the study are publicly available and are cited in the reference section.

## Supplementary Materials

The following supporting information can be downloaded at https://www.mdpi.com/article/10.3390/vaccines12101197/s1, The supplementary material provides a full list of model inputs and their source references (Table S1). References [60,61,62,63,64,65,66,67,68,69,70,71,72,73,74,75,76] are cited in the supplementary materials.
