# Supplementary material for: Burden of Pneumococcal Disease in Young Children Due to Serotypes Contained in Different Pneumococcal Conjugate Vaccines in Eight Asian Countries and Territories
Source: Vaccines (Basel). 2024 Oct 19;12(10):1197. doi: 10.3390/vaccines12101197 (PMC11511336; doi:10.3390/vaccines12101197)
Supplement: Supplementary file 1 [file vaccines-12-01197-s001.zip › vaccines-3266313-supplementary.pdf]

Supplementary material

Suppl. Table S1. Model inputs

	Hong Kong	Singapore	Taiwan	Thailand	Malaysia	Philippines	India	Indonesia
**Population aged 0-4 y**	228,994[60]	178,085[12]	811,733[61]	2,275,366	2,612,247[62]	11,064,672	346,149,367	22,414,317[63]
**Incidence/100,000/y**								
IPD	8.5[29]	4.3[12]	1.66[64]	12.46[65]	45[24]	128[49]	17.8[66]	80[67]
All-cause PNE	478.03[22,68]	1,120[22,69]	3,421[22]	3,627[23]	6,467[24,70,71]	5,220[22,49]	7,517[20,26]	25,000[25]
AOM	16,746[71]	8,522[22]	8,522[22]	601[23]	21,179[24]	9,570[49]	3,220[26]	11,270[72]
**Proportion of PNE cases pneumococcal (%)**	18[20]	18[20]	18[20]	18[20]	18[20]	18[20]	18[20]	18[20]
**Proportion of hospitalized PNE (%)**	21[22]	21[22]	21[22]	67.4[23]	39.1[24]	21[22]	43[26]	8[25]
**Proportion of non-hospitalized PNE (%)**	79[22]	79[22]	79[22]	32.6[23]	60.9[24]	79[22]	57[26]	92[25]
**Proportion of pneumococcal AOM (%)**	26.4[21]	26.4[21]	26.4[21]	26.4[21]	26[21]	26.4[21]	26[21]	26[21]
**Case fatality rate (%)**								
IPD	9[71]	6.8[69]	2.4[22]	20[23]	25.14[24]	37.5*	7[26]	57[67]
Hospitalized PNE	0.2[71]	0.6[69]	0.1[22]	1.4[23]	4[24]	12.08[73]	3[26]	1.8[74]
**Direct medical cost per case (USD)**								
IPD	14,671[71]	10,772[69]	2,462[22]	1,560[23]	4,181[75]	399[50]	590[65]	851[67]
Hospitalized PNE	7,189[71]	9,781[69]	465[22]	501[23]	3,123[75]	249[50]	328[65]	1191[67]
PNE GP visits	107[71]	1,721[69]	16[22]	7[23]	515[75]	2[50]	42[65]	31[67]
AOM	33[71]	107[69]	15[22]	11[23]	566[75]	118[50]	3[76]	31[67]

AOM, acute otitis media; GP, general practitioner; IPD, invasive pneumococcal disease; PNE, pneumonia

*Calculated value

References

Note: reference numbers correspond to those in the reference list of the main text

7. Active Bacterial Core Surveillance (ABCs) Report, Emerging Infections Program Network *Streptococcus pneumoniae*, 2019: Centers for Disease Control and Prevention, (2019).
12. Ministry of Health: Communicable Diseases Division. Communicable Diseases Surveillance: SINGAPORE 2018, (2019).
20. Lansbury L, Lim B, McKeever TM, Lawrence H, Lim WS. Non-invasive pneumococcal pneumonia due to vaccine serotypes: A systematic review and meta-analysis. *EClinicalMedicine* 2022;44:101271.
21. Ngo CC, Massa HM, Thornton RB, Cripps AW. Predominant bacteria detected from the middle ear fluid of children experiencing otitis media: A systematic review. *PLoS One* 2016;11(3):e0150949.
22. Lu CY, Chung CH, Huang LM, et al. Cost-effectiveness evaluation of the 10-valent pneumococcal non-typeable Haemophilus influenzae protein D conjugate vaccine for children in Taiwan. *Cost Effectiveness and Resource Allocation* 2020;18:30.
23. Dilokthornsakul P, Kengkla K, Saokaew S, et al. An updated cost-effectiveness analysis of pneumococcal conjugate vaccine among children in Thailand. *Vaccine* 2019;37(32):4551-60.
24. Shafie AA, Ahmad N, Naidoo J, et al. Estimating the population health and economic impacts of introducing a pneumococcal conjugate vaccine in Malaysia- an economic evaluation. *Human Vaccines & Immunotherapeutics* 2020;16(7):1719-27.
25. Oktaria V, Danchin M, Triasih R, et al. The incidence of acute respiratory infection in Indonesian infants and association with vitamin D deficiency. *PLOS ONE* 2021;16(3):e0248722.
26. Krishnamoorthy Y, Eliyas SK, Nair NP, Sakthivel M, Sarveswaran G, Chinnakali P. Impact and cost effectiveness of pneumococcal conjugate vaccine in India. *Vaccine* 2019;37(4):623-30.
29. Ho PL, Law PY, Chiu SS. Increase in incidence of invasive pneumococcal disease caused by serotype 3 in children eight years after the introduction of the pneumococcal conjugate vaccine in Hong Kong. *Human Vaccine & Immunotherapeutics* 2019;15(2):455-8.
49. Reassessment of 10- versus 13-valent Pneumococcal Conjugate Vaccines (PCV) in the Philippines: Republic of the Phillipines DOH Health Technology Assessment Unit, (2020).
50. Perdrizet J, Horn EK, Nua W, et al. Cost-effectiveness of the 13-valent pneumococcal conjugate vaccine (PCV13) versus lower-valent alternatives in filipino infants. *Infectious Diseases and Therapy* 2021;10(4):2625-42.
60. Government of the Hong Kong Special Administrative Region. 2021 Population Census (2021). https://www.censtatd.gov.hk/en/scode600.html (accessed 19 January 2024).
61. Government Portal of the Republic of China (Taiwan). Population Estimate Enquiry System (2022). https://pop-proj.ndc.gov.tw/ (accessed 19 January 2024).
62. World Bank. Population, total - Malaysia. https://data.worldbank.org/indicator/SP.POP.TOTL?locations=MY (accessed 19 January 2024).

63. World Bank. Population estimates and projections. https://databank.worldbank.org/source/population-estimates-and-projections/Type/TABLE/preview/on (accessed 19 January 2024).
64. Taiwan Centers for Disease Control. Available online: https://www.cdc.gov.tw/En/Professional/ProgramResultInfo/ppxd4Xu5zcYwcLHniXKk6w?programResultId=G89T_U6J5FcoUujUJ0hXwQ (Accessed 19 January 2024)
65. Pfizer Inc. Data on File. 2023. Available online: https://www.pfizer.com/ (accessed on 19 January 2024).
66. Nisarga R, Premalatha R, Shivananda, et al. Hospital-based surveillance of invasive pneumococcal disease and pneumonia in South Bangalore, India. *Indian Pediatrics* 2015;52(3):205-11.
67. Suwantika AA, Zakiyah N, Abdulah R, et al. Cost-effectiveness and budget impact analyses of pneumococcal vaccination in Indonesia. *Journal of Environmental and Public Health* 2021;2021:7494965.
68. Yu Q, Li X, Fan M, et al. The impact of childhood pneumococcal conjugate vaccine immunisation on all-cause pneumonia admissions in Hong Kong: A 14-year population-based interrupted time series analysis. *Vaccine* 2021;39(19):2628-35.
69. Tyo KR, Rosen MM, Zeng W, et al. Cost-effectiveness of conjugate pneumococcal vaccination in Singapore: Comparing estimates for 7-valent, 10-valent, and 13-valent vaccines. *Vaccine* 2011;29(38):6686-94.
70. Aljunid S, Abuduxike G, Ahmed Z, Sulong S, Nur AM, Goh A. Impact of routine PCV7 (Prevenar) vaccination of infants on the clinical and economic burden of pneumococcal disease in Malaysia. *BMC Infectious Diseases* 2011;11(1):248.
71. Wu DB, Roberts C, Lee VW, et al. Cost-effectiveness analysis of infant universal routine pneumococcal vaccination in Malaysia and Hong Kong. *Human Vaccines & Immunotherapeutics* 2016;12(2):403-16.
72. Anggraeni R, Carosone-Link P, Djelantik B, et al. Otitis media related hearing loss in Indonesian school children. *International Journal of Pediatric Otorhinolaryngology* 2019;125:44-50.
73. Haasis MA, Ceria JA, Kulpeng W, Teerawattananon Y, Alejandria M. Do pneumococcal conjugate vaccines represent good value for money in a lower-middle income country? A cost-utility analysis in the Philippines. *PLoS One* 2015;10(7):e0131156.
74. Azmi S, Aljunid SM, Maimaiti N, et al. Assessing the burden of pneumonia using administrative data from Malaysia, Indonesia, and the Philippines. *International Journal of Infectious Diseases* 2016;49:87-93.
75. Aljunid S, Maimaiti N, Ahmed Z, et al. Economic impact of pneumococcal Protein-D Conjugate Vaccine (PHiD-CV) on the Malaysian National Immunization Programme. *Value in Health Regional Issues* 2014;3:146-55.
76. Pfizer Inc. Data on File. 2024. Available online: https://www.pfizer.com/ (accessed on 19 January 2024).
